# Polyamines delay leaf maturation in low‐alkaloid tobacco varieties

**DOI:** 10.1002/pld3.77

**Published:** 2018-07-31

**Authors:** Greta Nölke, Daniel Volke, Ivana Chudobová, Marcel Houdelet, Marcos Lusso, Jesse Frederick, Andrew Adams, Chengalrayan Kudithipudi, Ujwala Warek, James A. Strickland, Dongmei Xu, Helga Schinkel, Stefan Schillberg

**Affiliations:** ^1^ Fraunhofer Institute for Molecular Biology and Applied Ecology IME Aachen Germany; ^2^ Altria Client Services Research Development & Sciences Richmond Virginia

**Keywords:** ethylene, inhibition of biosynthesis, maturation, *nic1*/*nic2* mutation, nicotine, ornithine decarboxylase, senescence

## Abstract

The development of low‐alkaloid (LA) tobacco varieties is an important target in the tobacco breeding industry. However, LA Burley 21 plants, in which the *Nic1* and *Nic2* loci controlling nicotine biosynthesis are deleted, are characterized by impaired leaf maturation that leads to poor leaf quality before and after curing. Polyamines are involved in key developmental, physiological, and metabolic processes in plants, and act as anti‐senescence and anti‐ripening regulators. We investigated the role of polyamines in tobacco leaf maturation by analyzing the free and conjugated polyamine fractions in the leaves and roots of four Burley 21 varieties: NA (normal alkaloid levels, wild‐type control), HI (high intermediates, *nic2* ^−^), LI (low intermediates, *nic1* ^−^), and LA (*nic1* ^−^ *nic2* ^−^). The pool of conjugated polyamines increased with plant age in the roots and leaves of all four varieties, but the levels of free and conjugated putrescine and spermidine were higher in the LI and LA plants than NA controls. The increase in the polyamine content correlated with delayed maturation and senescence, i.e., LA plants with the highest polyamine levels showed the most severe impaired leaf maturation phenotype, characterized by higher chlorophyll content and more mesophyll cells per unit leaf area. Treatment of LA plants with inhibitors of polyamine biosynthesis and/or the growth regulator Ethephon^®^ reduced accumulation of polyamines, achieving a partial amelioration of the LA phenotype. Our data show that the regulation of polyamine homeostasis is strongly disrupted in LA plants, and that free and conjugated polyamines contribute to the observed impairment of leaf maturation.

## 1 INTRODUCTION

Tobacco is one of the most widely grown non‐food crops in the world with global production of 6.6 million tons in 2016 (https://www.statista.com/statistics/261189/global-tobacco-production-since-1980/) and resulting tobacco products having an annual global market size of USD 770 billion (Euromonitor International, 2016). Despite awareness of the potential health risks and economic consequences associated with the use of tobacco products, more than 1.1 billion people worldwide smoked in 2015 (WHO, 2015). The widespread use of tobacco products can be somewhat related to the stimulatory and addictive effects of nicotine, the main alkaloid accumulating in tobacco leaves. Nicotine and other minor alkaloids are also precursors to tobacco‐specific nitrosamines (TSNA). As some TSNAs have been classified as human carcinogens, many in public health consider TSNAs as a factor in tobacco product use related cancers (Hecht, 2003; IARC, 2007). Tobacco addiction is one of the biggest public health issues and is responsible for more than 7 million deaths per year worldwide, of which 6 million deaths are related to direct use of tobacco (WHO, 2015). The negative health impact and high economic costs of smoking, and pending government regulations to control tobacco products, have fueled demands for development of tobacco cultivars with lower levels of nicotine, which would result in less addictive tobacco products with lower TSNA levels. It has been postulated that lower nicotine content in tobacco products would lead to a substantial reduction in tobacco‐related mortality (Apelberg et al., 2018). This is the reason why the FDA issued an advanced notice of proposed rulemaking to guide the development of a potential nicotine product standard that would reduce nicotine content in combusted cigarettes to minimally or non‐addictive levels (://www.federalregister.gov/documents/2018/03/16/2018-05345/tobacco-product-standard-for-nicotine-level-of-combusted-cigarettes).

In commercial tobacco cultivars, nicotine represents 90%–95% of the total alkaloid pool or 2%–5% of total leaf dry weight (Saitoh, Nona, & Kawashima, 1985). Nicotine is synthesized in the roots (Dawson, 1942), and translocated through the xylem (Baldwin, 1988) to aerial parts of the plant (Hildreth et al., 2011) where it accumulates and is stored in the vacuole of mesophyll leaf cells (Saunders, 1979), and is exuded by trichomes in response to insect herbivory (Kessler & Baldwin, 2002). Nicotine biosynthesis is influenced by genetic factors, plant development, biotic and abiotic stresses, phytohormonal signals, and agronomic management practices such as topping and suckering (Shoji & Hashimoto, 2015; Wang et al., 2008). The genetic regulation of nicotine biosynthesis correlates to two independent loci, *Nic1* and *Nic2*, which have a synergistic effect on nicotine levels, but the effect of *Nic1* is ~2.4 times stronger than that of *Nic2* (Legg & Collins, 1971). Both loci also influence the expression of numerous other genes unrelated to the nicotine biosynthesis pathway (Kidd et al., 2006; Shoji, Kajikawa, & Hashimoto, 2010). Transcriptional analysis has shown that the *Nic2* locus is a gene cluster that encodes at least seven ethylene response transcription factors (ERFs; Shoji et al., 2010), whereas characterization of the *Nic1* locus has not been fully disclosed.

The homozygous deletion of either one or both loci has been used to create near‐isogenic Burley 21 lines with reduced alkaloid levels, i.e., a high‐intermediate (HI) variety with the genotype *nic2* ^*−*^, a low‐intermediate (LI) variety with the genotype *nic1* ^*−*^, and a low‐alkaloid (LA) variety with the genotype *nic1* ^*−*^ *nic2* ^*−*^ (Legg, Chaplin, & Collins, 1969; Legg & Collins, 1971). LA Burley 21 plants contain only ~5.7% of the total alkaloid levels found in the normal‐alkaloid (NA) wild‐type variety (Legg, Collins, & Littion, 1970). In LA plants, the synergistic effect of the *nic1* ^*−*^ and *nic2* ^*−*^ deletions also causes an unfavorable leaf phenotype characterized by lower yields, delayed ripening and senescence, higher susceptibility to insect herbivory, and poor end‐product quality after curing (Chaplin & Burk, 1983; Chaplin & Weeks, 1976; Legg et al., 1970). A better understanding of the differences between LA and NA plants could facilitate strategies to improve the quality of LA leaf and its resulting tobacco products and allow the development of new tobacco cultivars with lower alkaloid levels combined with comparable or superior product characteristics.

Nicotine comprises a pyrrolidine ring and a pyridine ring, each of which are generated via an independent primary metabolic pathway (Dewey & Xie, 2013; Katoh & Hashimoto, 2004). The pyrrolidine ring is derived from the ubiquitous polyamine putrescine, which is also a key intermediate in the synthesis of the higher polyamines spermidine and spermine. In plants, polyamines are involved in key developmental, physiological, and metabolic processes such as cell growth and division, stress tolerance, vascular differentiation, lignin polymerization, pathogen defense, senescence, and ripening (Fariduddin, Varshney, Yusuf, & Ahmad, 2013; Kusano & Suzuki, 2015). Several studies have linked polyamines to the regulation of plant cell senescence, although the detailed mechanism is not yet known (Sobieszczuk‐Nowicka, Kubala, Zmienko, Malecka, & Legocka, 2016). In fruit and vegetative tissues, polyamines act as anti‐senescence and anti‐ripening regulators that prevent the decay of chloroplast photosystem complexes and changes in cell wall/membrane composition (Lester, 2000; Mattoo & Handa, 2008; Serafini‐Fracassini, Di Sandro, & Del Duca, 2010). Higher levels of polyamines increase the longevity of tomato vines (Mehta et al., 2002), and delayed ripening and leaf senescence was observed in transgenic tomato plants overexpressing a yeast spermidine synthase (Nambeesan et al., 2010). Polyamines may act directly by stabilizing cell walls or through crosstalk with phytohormones such as ethylene, abscisic acid, cytokinins, and gibberellins (Kusano & Suzuki, 2015).

In most plants, putrescine can be synthesized either directly from ornithine by ornithine decarboxylase (ODC) or from arginine via three enzymatic steps, initiated by arginine decarboxylase (ADC; Illingworth et al., 2003; Michael, Furze, Rhodes, & Burtin, 1996; Piotrowski, Janowitz, & Kneifel, 2003). Previous studies have demonstrated that the ADC route to putrescine has only a minor effect on the alkaloid profile of tobacco whereas the ODC pathway plays the major role in nicotine biosynthesis (Chintapakorn & Hamill, 2007; Dalton et al., 2016; DeBoer, Dalton, Edward, & Hamill, 2011; DeBoer, Dalton, Edward, Ryan, & Hamill, 2013). Putrescine is converted to spermidine and then spermine by the successive addition of aminopropyl groups derived from decarboxylated S‐adenosylmethionine (SAM), in reactions catalyzed by the enzymes spermidine synthase and spermine synthase, respectively. SAM is also a substrate for the biosynthesis of ethylene (Tiburcio, Altabella, Bitrián, & Alcázar, 2014), which regulates senescence and fruit ripening (Fluhr & Mattoo, 1996). The polyamine and ethylene biosynthesis pathways compete for the common precursor SAM but have opposing developmental effects, particularly during the developmental switch from vegetative growth to ripening/senescence (Gupta, Pal, & Rajam, 2013; Harpaz‐Saad, Yoon, Mattoo, & Kieber, 2012; Nambeesan, Handa, & Mattoo, 2008). Polyamine levels decrease and ethylene levels increase during the onset of fruit ripening in tomato (Morilla, Garcia, & Albi, 1996; Saftner & Baldi, 1990) and avocado (Kushad, Yelenosky, & Knight, 1988), which reflects the mutually antagonistic effect of ethylene on polyamine biosynthesis and vice versa (Anwar, Mattoo, & Handa, 2015; Harpaz‐Saad et al., 2012). However, transgenic tomato plants expressing yeast S‐adenosylmethionine decarboxylase (SAMDC) under the control of the ripening‐specific *E8* promoter produced higher levels of ethylene and polyamines simultaneously during fruit ripening, indicating the absence of any competition for SAM in this system (Mehta et al., 2002).

We hypothesized that the suppression of nicotine biosynthesis in LA tobacco plants would affect crosstalk between the nicotine, polyamine, and ethylene pathways, resulting in the accumulation of putrescine. This would in turn increase metabolic flux toward the higher polyamines spermidine and spermine while inhibiting ethylene biosynthesis, causing a dramatic effect on leaf maturation and senescence. We evaluated this hypothesis by investigating the impact of polyamines on the undesirable leaf phenotype of LA plants. We measured the polyamine content in the roots and leaves of NA, HI, LI, and LA Burley 21 lines and determined the relationship between polyamine levels, leaf morphology and growth. We also exposed LA plants to polyamine inhibitors and the growth regulator Ethephon^®^ (2‐chloroethylphosphonic acid), and evaluated the effect of these treatments on the leaf phenotype compared to wild‐type NA plants with desirable leaf characteristics.

## 2 MATERIAL AND METHODS

### 2.1 Plant material and growth conditions

Seeds of *Nicotiana tabacum* L. cv. Burley 21 wild‐type NA, as well as HI (*nic2* ^*−*^), LI (*nic1* ^*−*^) and LA (*nic1* ^*−*^ *nic2* ^*−*^) near‐isogenic varieties were obtained from the US Nicotiana Germplasm Collection at North Carolina State University and used in all greenhouse experiments. Seeds were germinated in pots under greenhouse conditions at 27/23°C day/night temperature and a 16‐hr photoperiod (~200 mmol s^−1^ m^−2^; *λ* = 400–700 nm) at 70% relative humidity. Five‐week‐old tobacco plantlets were transferred to 13‐L pots with standard substrate (Einheitserde, Fröndenberg, Germany). The plants were attached to a continuous drip irrigation system active every 4 hr for ~5 min and were irrigated with 0.7% (w/v) Ferty 2 Mega containing 16% nitrogen (Planta Düngemittel, Regenstauf, Germany) during the 16‐hr photoperiod and grown for 4 additional weeks. Greenhouse plants were topped when 50% of the plants had at least one open flower. After topping, plants grew until harvest, 30 days post‐topping. Tobacco leaves used for polyamine analysis were collected from greenhouse‐grown plants at three time points: before flowering (6.5‐week‐old plants), just before topping, i.e., the removal of the floral apex (9‐week‐old plants) and at harvest (13‐week‐old plants, 4 weeks post‐topping). Root samples were collected at topping and harvest. In 2015, LA and NA tobacco plants were grown in the field in Virginia under 135 units of nitrogen per acre and sampled at 1 week post‐topping for polyamine analysis.

For treatment with polyamine biosynthesis inhibitors, 5 mM d‐arginine (AKos, Steinen, Germany), 2 mM difluoromethylornithine (DFMO) (Synchem Ug & Co. KG, Felsberg, Germany) alone or in combination with 0.5 mM Ethephon^®^, or 0.5 mM Ethephon^®^ alone (Merck KGaA, Darmstadt, Germany), were diluted in the same amount of water used for daily irrigation and applied to LA plants every 4 hr at 9 a.m., 12 a.m., 3 p.m., and 6 p.m. three times per week instead of the drip irrigation system. The treatment with d‐arginine and DFMO started before flowering (~2.5 weeks before topping when plants were still in the vegetative growth stage) for a period of 6 weeks until harvest, whereas Ethephon^®^ was applied from topping to harvest (4 weeks in total) to avoid early senescence in the LA plants. Twelve plants per inhibitor were treated. Non‐treated NA plants and non‐treated LA plants were used as controls.

### 2.2 Chlorophyll measurements

The chlorophyll content was determined by measuring leaf absorbance in the red and infrared regions using a SPAD‐502 Plus device (Minolta Camera Co., Osaka, Japan). Chlorophyll was measured twice in the same day at different positions in all fully expanded (length > 15 cm) leaves (leaves 6–26) from six randomly‐selected plants from each line at five growth stages: before flowering (6.5‐week‐old plants and 2.5 weeks before topping), at topping, 1 and 2.5 WPT and at harvest (4 WPT). The total chlorophyll content was calculated as an average of all measured leaf chlorophyll values per plant to minimize the influence of leaf position.

### 2.3 Leaf cell microscopy

Four leaf disks (1 cm^2^) cut from leaf 15 from six biological replicates at different development stages (before flowering, at topping, 1 WPT and at harvest) were mounted on slides and imaged using a Leica DM R microscope (Leica, Wetzlar, Germany) with a 10× air objective. Images were imported into ImageJ and Adobe Photoshop CS5.1 software and the cells per unit area were counted using Count Tool in the Photoshop CS5. A standard area was designated to use for cell counting three times across all images and care was taken to avoid counting any cell twice.

### 2.4 Determination of ODC and ADC activities

To determine enzymatic activities, 500 mg of tobacco leaf tissue collected from leaf 23 of three biological replicates of NA and LA plants was ground in 1 ml HEPES extraction buffer (100 mM HEPES, 2 mM dithiothreitol (DTT), 1 mM EDTA, pH 7.5) and 100 mg of polyvinylpyrrolidone was added during grinding. Following centrifugation (13,000 *g*, 10 min, 4°C), the enzyme activities were measured using an isotopic method as described by Capell et al. (1998) by measuring the release of ^14^CO_2_. DL‐[1‐^14^C]Arg and L‐[1‐^14^C]Orn (American Radiolabeled Chemicals, St Louis, USA) were used as radioactive substrates.

### 2.5 Polyamine extraction and analysis

For polyamine analysis, 150 μg of leaf or root material was harvested from plants grown in the greenhouse at different stages of development: before flowering (leaves 6 and 12, numbered from base), at topping (leaves 19 and 23 and roots), and at harvest (leaves 23 and 24 and roots). Samples were collected after 4 hr of illumination from three biological replicates and were flash frozen in liquid nitrogen. For field‐grown plants, leaf material was collected from five well‐expanded upper leaves from three biological replicates. Plant material was ground in 1.6 ml pre‐chilled 10% (v/v) perchloric acid and incubated at 4°C for 1 hr. The extract was vortexed for 10 s and centrifuged (16,000 *g*, 15 min, 4°C) before 800 μl of the supernatant was mixed with 100 μl 1 mM hexamethylenediamine. Then, 10 μl of the clear supernatant was transferred to a fresh 2‐ml tube and polyamines were extracted with 200 μl of cyclohexane for the dansilation of free polyamines. For the extraction of conjugated polyamines, the pellet was resuspended in 1,600 μl 1 M NaOH and 200 μl 1 mM hexamethylenediamine and centrifuged as above. The clear supernatant (200 μl) was transferred to a 2‐ml glass ampule containing 12 M HCl, mixed and incubated for 16 hr at 110°C overnight for the hydrolysis of conjugated polyamines. The dansilation of free and conjugated polyamines was carried out with dansyl chloride as described by Flores and Galston (1982).

The dansylated polyamines were measured by LC‐MS/MS. All experiments were carried out on a 3200 QTRAP™ mass spectrometer (Sciex, Darmstadt, Germany) coupled to an HPLC Agilent 1200 system (Waldbronn, Germany). The mass spectrometer was equipped with an electrospray ionization source. The sample was separated on a reversed‐phase Synergi Fusion with 80 Å pore size, 4 μm particle size, and dimensions of 50 mm × 2.0 mm internal diameter (Phenomenex, Aschaffenburg, Germany) with the corresponding guard column at a flow rate of 800 μl/min. The column oven was heated to 30°C. For elution, solvent A comprised 94.9% (v/v) water, 5% (v/v) acetonitrile, 0.1% (v/v) formic acid and solvent B comprised 94.9% (v/v) acetonitrile, 5% (v/v) water, 0.1% (v/v) formic acid. The elution following elution profiles was used: 1 min, hold at 60% solvent A/40% solvent B; 3 min, linear increase to 100% solvent B, 3 min hold at 100% solvent B; rapid linear decrease to 60% solvent A/40% solvent B in 0.1 min; hold for 1 min. The total run time was 8 min and the sample volume injected in each run was 10 μl.

The mass spectrometer was set to unit resolution in Q1 and Q3. All measurements were captured in multiple reaction monitoring mode. For compound optimization, standards were prepared according to the dansylation protocol, diluted in 50:50 (v/v) methanol/water, and infused with a flow rate of 10 μl/min with the syringe pump directly connected to the ion source. Declustering potential, collision energy, collision cell entrance potential, collision cell exit potential, and entrance potential were optimized for all compounds using automated compound optimization (Supporting Information Table S1). The ion source parameters were set to: capillary voltage = 5.5 kV, heater gas temperature = 500°C, curtain gas = 30 psi, nebulizing gas = 70 psi, drying gas = 70 psi, and collision gas = medium. For each analyte, one transition was used for quantification and another as a qualifier. The acquired data were processed using Analyst v1.6 (Sciex). The mass calibration of the 3200 QTRAP was achieved using polypropylene glycol standards (Standards Chemical Kit with Low/High Concentration PPGs, Sciex) according to the manufacturer's instructions.

### 2.6 Statistical analysis

Significant differences between the genotypes were determined by applying one‐way analysis of variance (ANOVA) followed by post hoc Bonferroni test using Excel software (Microsoft, Redmond, Washington, USA). Two‐tailed t‐tests were applied. A *p*‐value <0.05 was considered statistically significant.

## 3 RESULTS

### 3.1 Biochemical and morphological differences among the four varieties during leaf maturation

**Figure 1:**
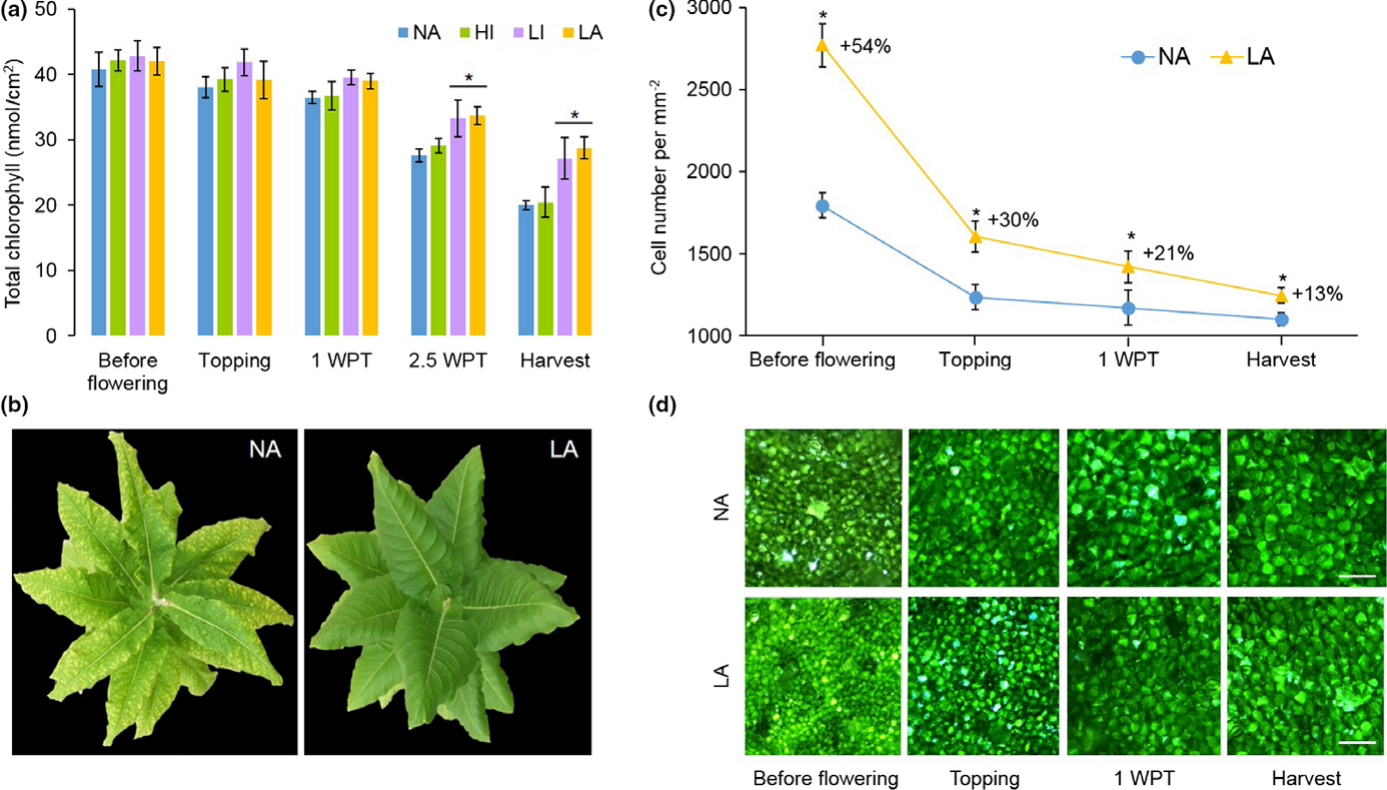
Phenotypic characterization of *Nicotiana tabacum* L. cv. Burley 21 normal‐alkaloid (NA) wild‐type plants and three mutant varieties grown in the greenhouse. (a) Total chlorophyll content of wild‐type (NA) high‐intermediate (HI, *nic2* ^*−*^), low‐intermediate (LI, *nic1* ^*−*^), and low‐alkaloid (LA, *nic1* ^*−*^ *nic2* ^*−*^) lines grown in the greenhouse. Chlorophyll was measured twice per leaf in all leaves longer than 15 cm at different developmental stages: before flowering (2.5 weeks before topping), at topping, 1‐week post‐topping (WPT), 2.5 WPT and harvest. Values are means of four biological replicates. (b) Representative photos of NA and LA plants at harvest. (c) Time‐course evaluation of mesophyll cell number in leaf 15. (d) Microscopic images of mesophyll cells of leaf 15 from NA and LA plants at different plant developmental stages. Bar = 100 μm. Values in A and C are means of six biological replicates. Error bars represent standard deviations of the mean. Statistical difference to NA is shown: *p < 0.05

The progression of senescence in the Burley 21 NA, HI, LI, and LA lines was monitored by measuring the loss of chlorophyll *a* and *b* in the leaves. The chlorophyll levels had declined significantly (*p* < 0.01) in all genotypes after 1‐week post‐topping (WPT; Figure 1a). However, the leaves of the LI and LA plants contained significantly (*p* < 0.001) higher levels of chlorophyll than the NA controls at 2.5 WPT (22% more in both genotypes) and at harvest (36% and 44% more in the LI and LA leaves, respectively), indicating slower chlorophyll degradation compared to NA controls. The loss of chlorophyll was correlated with morphological changes in the leaves of NA plants, i.e., they became wrinkly and leathery with yellow patches, whereas the LA leaves remained smooth, shiny, and green (Figure 1b).

Given the distinct leaf morphology in the LA and NA lines, we investigated the size and shape of the mesophyll cells at different time points. Before flowering, leaf 15 (numbered from the base) of the LA plants had smaller and more abundant mesophyll cells (more cells per unit leaf area) compared to the NA plants (Figure 1c,d). From that time point until harvest, the number of leaf mesophyll cells per unit area declined at a similar rate in both the NA and LA lines, but the LA plants retained a significantly (*p* < 0.05) greater number of mesophyll cells throughout maturation. The greatest difference in mesophyll cell number per unit area (54% more cells in the LA plants compared to NA controls) was observed at earlier stages of leaf development (before flowering). The LI plants also contained more mesophyll cells than the NA plants but not to the degree observed in the LA plants, and there was no significant difference in mesophyll cell number between the HI and NA lines (data not shown).

### 3.2 LA plants accumulate higher levels of polyamines than NA plants

**Figure 2:**
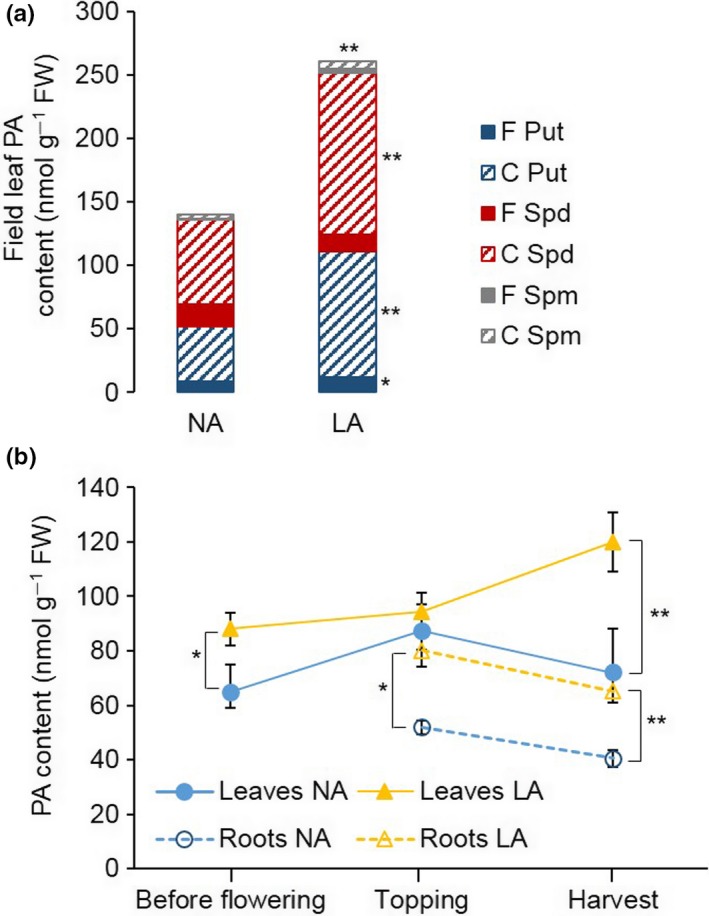
Polyamine analysis in field‐ and greenhouse‐grown NA and LA plants. (a) Free (F) and conjugated (C) putrescine (Put), spermidine (Spd), and spermine (Spm) content from five well‐expanded upper leaves of NA and LA plants (three biological replicates grown in the field) 1 week after topping. (b) Time‐course monitoring of total polyamine content in NA and LA plants grown in the greenhouse. Leaf samples were collected from leaves at the same stage of development: before flowering (leaf 12), at topping (leaf 19), and at harvest (leaf 24). Root samples were collected at topping and harvest. Values are means of three (a)/four (b) biological replicates. Error bars represent standard deviations of the mean. Statistical difference to NA is shown: *p < 0.05; **p < 0.005. PA: polyamine; FW: fresh weight

To investigate the impact of the *nic1* ^*−*^ *nic2* ^*−*^ double deletion on polyamine biosynthesis, we compared the levels of free and conjugated putrescine, spermidine and spermine in the NA and LA plants by liquid chromatography tandem mass spectrometry (LC‐MS/MS). First, we analyzed the polyamine content in leaves 16–18 of field‐grown plants. At 1 WPT, the total polyamine content was significantly higher (1.9‐fold, *p* < 0.001) in the LA plants compared to the NA plants (Figure 2a). Compositional analysis revealed significantly higher levels of free putrescine (1.4‐fold, *p* < 0.05), conjugated putrescine (2.3‐fold, *p* < 0.005) and conjugated spermidine (1.9‐fold, *p* < 0.005) levels in the leaves of the LA plants, indicating that the polyamine biosynthesis pathway is strongly induced by the *nic1* ^*−*^ *nic2* ^*−*^ double deletion or that the inability of the substrates to be further processed into nicotine results in a buildup of these materials. In contrast, the level of free spermidine in the LA plants was lower than in the NA plants, although the difference was not statistically significant (*p* > 0.05).

To minimize the effect of variable environmental factors on polyamine biosynthesis, further experiments were performed under controlled greenhouse conditions mirroring the average field conditions in terms of temperature, light, and humidity (data not shown). The phenotypes of the NA and LA plants in the greenhouse at harvest (30 days post‐topping) were similar to their counterparts grown in the field in terms of plant height, leaf number, and leaf morphology (data not shown). The impact of wounding on polyamine biosynthesis was minimized by designing the experiments so that each leaf/root sample was collected only once per plant and time point.

Time‐course monitoring of the total polyamine content in leaves at the same developmental stage—i.e., leaf 12 before flowering, leaf 19 at topping, and leaf 24 at harvest—revealed significantly (*p* < 0.05) higher levels of polyamines in the LA plants before flowering (1.5‐fold) and at harvest (2.1‐fold) compared to the NA controls (Figure 2b). The LA plants also accumulated significantly (*p* < 0.05) higher levels of total polyamines in the roots at topping (1.5‐fold) and at harvest (1.4‐fold) compared to the NA controls (Figure 2b).

### 3.3 Effect of the *nic1* ^*−*^ *nic2* ^*−*^ double deletion on polyamine biosynthesis

**Figure 3:**
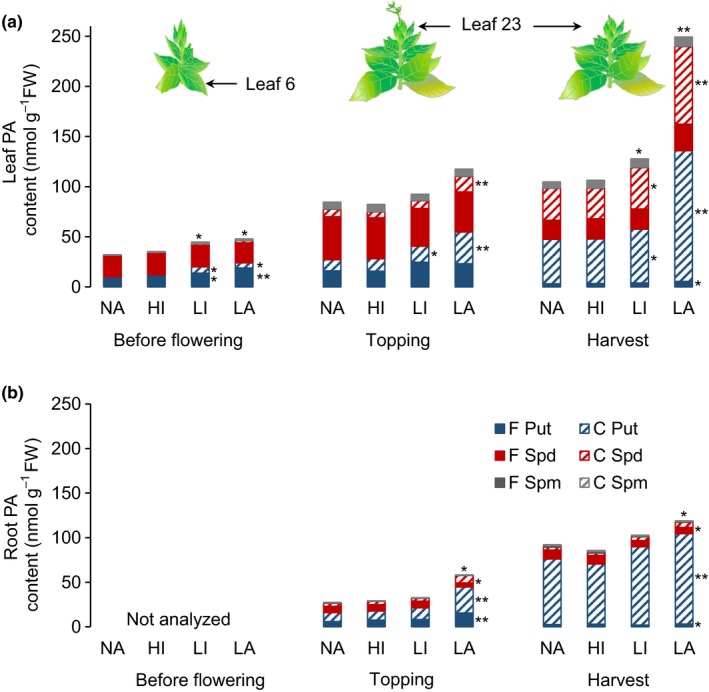
Polyamine content in the leaves and roots of NA, HI, LI, and LA plants grown in the greenhouse. (a) Free (F) and conjugated (C) putrescine (Put), spermidine (Spd) and spermine (Spm) fractions in leaves (a) and roots (b) of NA, HI, LI, and LA plants before flowering (leaf 6), at topping (young leaf 23, roots), and at harvest (matured leaf 23, roots). Samples were collected 4 hr after illumination, frozen immediately in liquid nitrogen and analyzed by LC‐MS/MS. Values are means of three biological replicates. Error bars represent standard deviations of the mean. Statistical difference is shown: *p < 0.05; **p < 0.001, indicating that the LI and LA plants are significantly different from NA plants under the same conditions. Only samples from topping and harvest were available for roots. FW: fresh weight

Comparative analysis of the polyamine composition in selected leaves (leaf 6 before flowering, young leaf 23 at topping, and mature leaf 23 at harvest) in the four varieties revealed that, before flowering, lines LI and LA contained significantly (*p* < 0.05) higher levels of free putrescine than the NA controls (1.6‐fold and 4.2‐fold higher, respectively) and even higher levels of conjugated putrescine (2.1‐fold and 5‐fold higher, respectively; Figure 3a). The conjugated putrescine and spermidine fractions increased continuously during leaf maturation in all four varieties, but remained significantly higher in LI and LA plants compared to NA controls (Figure 3a). The greatest differential in polyamine content was observed in the LA leaves at harvest, with a 2.1‐fold increase in the level of total polyamines compared to NA controls, including a 1.8‐fold increase in free putrescine, a 2.9‐fold increase in conjugated putrescine and a 2.4‐fold increase in conjugated spermidine. However, there was no significant difference between the NA and HI varieties, indicating that the single *nic2* ^*−*^ deletion had a lower impact on polyamine accumulation.

At topping, the roots of the LA plants contained significantly (*p* < 0.05) higher levels of free putrescine, conjugated putrescine, and conjugated spermidine than the NA plants (2.6‐fold, 2.9‐fold, and 2.5‐fold increases, respectively) and such differences were also observed at harvest (1.6‐fold, 1.4‐fold, and 2.5‐fold increases, respectively; Figure 3b).

### 3.4 The polyamine biosynthesis pathway is more active in the LA plants

**Figure 4:**
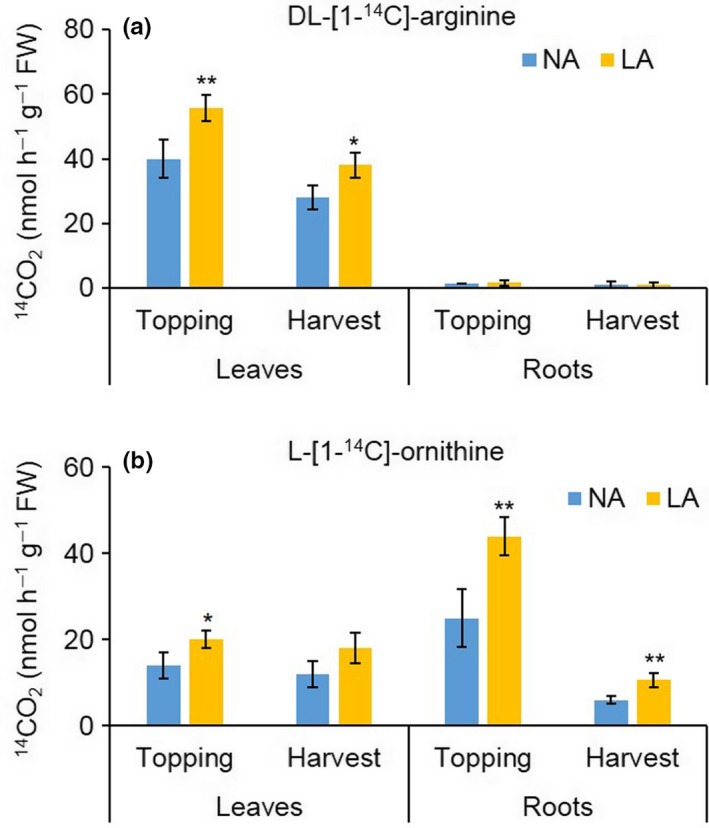
Activity of polyamine biosynthesis enzymes. Analysis of (a) arginine decarboxylase (ADC) and (b) ornithine decarboxylase (ODC) activity in leaves and roots of NA and LA plants at topping (young leaf 23, roots) and harvest (matured leaf 23, roots). Values are means of three biological replicates. Error bars represent standard deviations of the mean. Statistical difference to NA is shown: *p < 0.05; **p < 0.001

The relative contribution of ADC and ODC to putrescine biosynthesis was evaluated by measuring the activity of each enzyme in the leaves (leaf 23) and roots of the NA and LA plants at topping and harvest. ADC and ODC activity varied in an organ‐specific and developmental stage‐specific manner in both lines (Figure 4). Whereas ADC activity was high in the leaves but minimal in the roots of both lines, ODC activity was higher in the younger leaves and roots, indicating that ODC is mainly responsible for putrescine biosynthesis in the roots. ADC activity was significantly higher (1.4‐fold, *p* < 0.05) in the leaves of the LA plants compared to the NA controls at topping and harvest (Figure 4a). Similarly, ODC activity was significantly higher (*p* < 0.05) in the LA plants compared to the NA controls in the roots at topping (1.8‐fold) and at harvest (1.7‐fold), and in young leaves at topping (1.5‐fold; Figure 4b).

### 3.5 Inhibition of polyamine biosynthesis in the LA variety

Given the correlation between the higher polyamine levels in the LA variety and the undesirable leaf morphology, we evaluated the effect of treating the plants with chemicals that inhibit ADC and ODC. Preliminary experiments defined the appropriate inhibitor concentration, application time, treatment intensity and duration (data not shown). The levels of free and conjugated putrescine were significantly higher in the LA plants than the NA controls before flowering and at harvest (Figure 3), so we applied the ADC inhibitor d‐arginine and the ODC inhibitor DFMO beginning 2.5 weeks before topping and continued the treatment until harvest. In addition, the plant growth regulator Ethephon^®^ was used alone or in combination with DFMO to accelerate senescence via the liberation of ethylene. To avoid the early induction of senescence, Ethephon^®^ was applied from topping until harvest.

**Figure 5:**
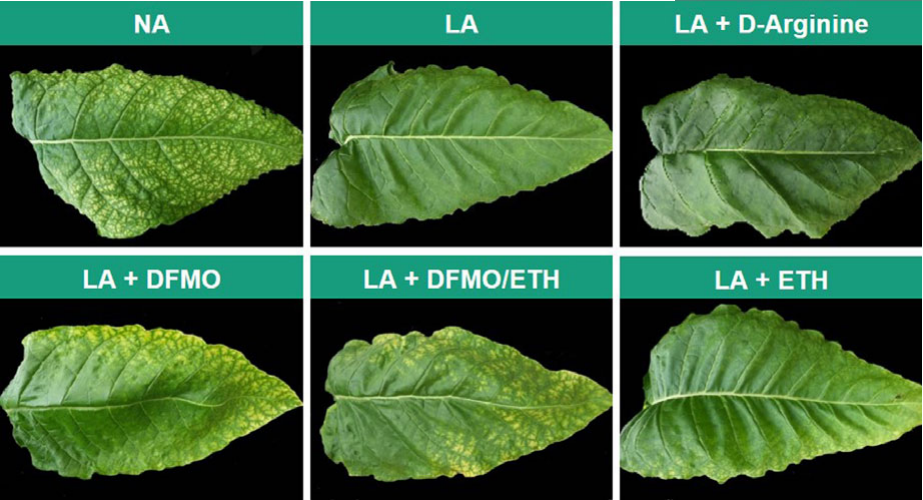
Representative photos of leaf 23 from untreated NA and LA plants and LA plants treated with polyamine biosynthesis inhibitors and/or plant growth regulator at harvest. d‐arginine (5 mM) is an inhibitor of ADC; DFMO (difluoromethylornithine, 2 mM) is an inhibitor of ODC, ETH (Ethephon^®^, 0.5 mM) is a growth regulator

The DFMO and DFMO/Ethephon^®^ treatments achieved a partial amelioration of the morphological phenotype, such that the leaves of the LA plants took on some of the characteristics of the NA leaves (wrinkling and chlorophyll degradation), whereas treatment with Ethephon^®^ alone reduced the chlorophyll content but did not affect leaf morphology (Figure 5). Starting the DFMO treatment before flowering resulted in growth arrest, which was not observed when the treatment was started at topping (data not shown). The d‐arginine treatment had no effect on the chlorophyll level or morphology of the LA plants.

**Figure 6:**
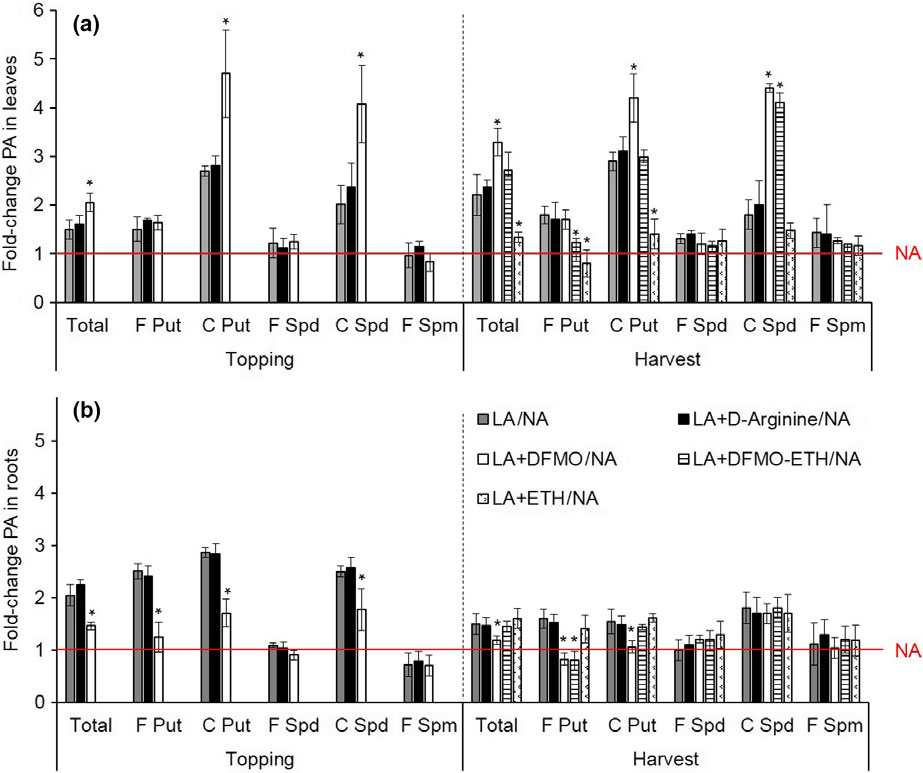
Treatment of LA plants with polyamine biosynthesis inhibitors and/or Ethephon^®^. Comparative total, free and conjugated polyamine in leaves (a) and roots (b) of untreated and treated LA plants at topping and harvest. Tobacco plants were grown in the greenhouse in the absence (NA and LA) or presence (LA) of polyamine biosynthesis inhibitors and/or Ethephon^®^ (5 mM d‐arginine, 2 mM DFMO, 2 mM DFMO/0.5 mM Ethephon^®^ or 0.5 mM Ethephon^®^ alone). d‐arginine and DFMO were applied three times per week from before flowering to harvest for a period of 6 weeks, whereas Ethephon^®^ treatment started after topping (2.5 weeks later) until harvest. Samples were collected 4 hr after illumination from leaf 23 or roots of four biological replicates per genotype or treatment. The fold change between the mean polyamine content from untreated LA plants (gray bars), or plants treated with d‐arginine (black bars), DFMO (white bars), DFMO/Ethephon^®^ (horizontal lined bars), and Ethephon^®^ (divot bars) are plotted. Error bars represent standard deviations of the mean (*n* = 4). Statistical difference to the mean of LA/NA is indicated: *p < 0.05. The red line represents polyamine content in NA

The analysis of polyamine levels revealed that the DFMO treatment 2.5 weeks before topping increased the levels of total polyamines in the LA leaves by 2.1‐fold, mainly reflecting higher levels of conjugated putrescine and conjugated spermidine (Figure 6a). This higher proportion of conjugated polyamines remained until harvest in the plants treated with DFMO and DFMO/Ethephon^®^. In contrast, the treatment with Ethephon^®^ alone led to a significant reduction in total polyamine levels at harvest, mainly reflecting the reduction of free and conjugated putrescine. In the roots, the DFMO treatment significantly reduced (*p* < 0.05) the total polyamine content of the LA plants at topping (1.5‐fold) and at harvest (1.4‐fold) due mainly to reduction of free and conjugated putrescine and conjugated spermidine (Figure 6b). This decrease was not reversed by the addition of Ethephon^®^. In contrast to the effect in leaves, the application of Ethephon^®^ alone had no effect on the polyamine content of the roots. The d‐arginine treatment had no effect on the polyamine content of the LA plants. The loss of polyamines in the roots could therefore reflect the inhibition of ODC activity, the main enzyme responsible for putrescine biosynthesis in roots (Figure 4).

## 4 DISCUSSION

Traditional breeding and molecular biotechnology approaches have been used to develop new tobacco varieties with lower nicotine and secondary alkaloid levels, but these LA varieties are characterized by impaired leaf maturation and senescence, resulting in an inferior end product. Assuming that the suppressed nicotine production in LA Burley 21 tobacco plants (*nic1* ^*−*^ *nic2* ^*−*^) affects the direct crosstalk between the nicotine, polyamine, and ethylene pathways, we evaluated the effect of polyamines on the development of the undesirable LA leaf phenotype. Changes in the levels of polyamines correlate with activation or repression of developmental response pathways such as ripening and senescence (Ioannidis et al., 2014; Mattoo & Handa, 2008; Sobieszczuk‐Nowicka, 2017). Whereas ethylene is a well‐known promoter of senescence, polyamines suppress this process by delaying membrane deterioration and the loss of chlorophyll while enhancing protease and RNase activities (Pandey, Ranade, Nagar, & Kumar, 2000; Sauter, Moffatt, Saechao, Hell, & Wirtz, 2013). The genes encoding diamine oxidase and polyamine oxidase are upregulated during leaf senescence (Ioannidis et al., 2014). The polyamine content must be tightly controlled with regard to end product (spermidine and spermine) accumulation to ensure normal plant development (Moschou & Roubelakis‐Angelakis, 2014; Moschou et al., 2012; Nölke, Schneider, Fischer, & Schillberg, 2005). Polyamine homeostasis is regulated by synthesis, transport to vacuoles, catabolism or interconversion, and conjugation with nucleic acids, membranes, proteins, or hydroxycinnamic acid (Ahmed et al., 2017; Bassard, Ullmann, Bernier, & Werck‐Reichhart, 2010; Chai, Yang, & Shi, 2017; Moschou & Roubelakis‐Angelakis, 2014).

The conjugation of polyamines with hydroxycinnamic acid to form the corresponding amides (HCAAs) reduces the abundance of free polyamines during senescence and controls the intracellular polyamine concentration (Bassard et al., 2010; Torras‐Claveria et al., 2012). Although numerous studies have linked the depletion of free polyamines to senescence (Cohen, Popovic, & Zalik, 1979; Serafini‐Fracassini et al., 2010), few reports have included an analysis of conjugated polyamines because they are more difficult to quantify. We measured the levels of both free and conjugated polyamines to provide a complete picture of polyamine homeostasis in the four Burley 21 varieties.

The total polyamine content of both field‐grown and greenhouse‐grown LA plants was higher than the corresponding NA controls, associated with delayed leaf maturation and senescence (Figures 2a and 3). Furthermore, the polyamine profile was shifted in the LA plants, with higher levels of conjugated putrescine and spermidine. The profile shift was more pronounced in field‐grown plants: the levels of conjugated putrescine and spermidine at 1 WPT in the field‐grown plants were similar to the levels measured in the greenhouse‐grown plants at harvest, suggesting that the field‐grown plants would accumulate even more conjugated polyamines by harvest. Given that these plants are exposed to a much greater biotic and abiotic stress, the elevated polyamine levels could reflects the important role of polyamines as modulator of stress response (Hussain, Ali, Ahmad, & Siddique, 2011). This agrees with previous studies showing that polyamines are higher in plants under both short‐term as well as long‐term abiotic and biotic stress conditions (Hussain et al., 2011; Minocha, Majumdar, & Minocha, 2014). The increase in the polyamine content correlated with the severity of the undesirable leaf phenotype. LA plants with the highest total polyamine content showed the most severe impairment of leaf maturation, characterized by weak senescence, a higher chlorophyll content, and more leaf mesophyll cells per unit area (Figure 1). Similarly, Ahmed et al. (2017) showed that significantly higher levels of conjugated putrescine, spermidine, and spermidine delayed senescence and the maturation of siliques in *Arabidopsis thaliana* plants constitutively expressing the polyamine transporter PUT.

Torras‐Claveria et al. (2012) reported lower levels of putrescine–hydroxycinnamic acid conjugates in tobacco leaves during senescence compared to non‐senescing leaves. Although we did not distinguish these specific conjugates from the pool of conjugated polyamines and did not analyze non‐senescing plants, the impaired leaf senescence in the LA Burley 21 variety and the increased abundance of conjugated polyamines supports this earlier study. Taken together, these data suggest that the regulation of polyamine homeostasis is strongly disrupted in the LA and LI varieties and that the higher total polyamine content plays a significant role in the development of the aberrant leaf phenotype in LA Burley 21 plants.

Young leaves in the LA variety were characterized by high ODC activity and a greater number of mesophyll cells per unit leaf area compared to NA controls, suggesting that the ODC activity stimulates rapid cell proliferation. This agrees with previous reports showing a positive correlation between ODC activity and cell division, and a negative correlation with cell expansion and cell size (Bertoldi, Tassoni, Martinelli, & Bagni, 2004; Paschalidis & Roubelakis‐Angelakis, 2005). ODC is the main enzyme responsible for putrescine biosynthesis in tobacco roots and plays a key role in the production of nicotine and the overall alkaloid profile (Chintapakorn & Hamill, 2007; Dalton et al., 2016; DeBoer et al., 2011, 2013). Topping of tobacco plants allows the crop to reach its full yield and quality potential at harvest and leads to increased activity of ODC and several enzymes in the nicotine biosynthesis pathway, including putrescine methyltransferase and quinolinate phosphoribosyltransferase, in tobacco roots 24–48 hr after topping (Mizusaki, Tanabe, Noguchi, & Tamaki, 1973; Saunders & Bush, 1979). Our LA Burley 21 plants had significantly higher ODC activity in the roots at topping and harvest (Figure 4b). Treatment with DFMO, an irreversible inhibitor for ODC, reduced the total polyamine levels in the roots of LA plants (Figure 6b) and partly ameliorated the leaf phenotype. Previous studies have shown that roots determine the status of aerial parts of the plant (Ruiz, Rios, Rosales, Rivero, & Romero, 2006; Ruiz, Rivero, Cervilla, Castellano, & Romero, 2006). When NA scion was grafted on LA rootstock the leaf and chemistry phenotype of the scion was consistent with the LA phenotype and when LA scion was grafted on NA rootstock, the leaf and chemistry phenotype of the scion was consistent with the NA phenotype (unpublished data). In leaves, where ADC rather than ODC is responsible for putrescine biosynthesis (Figure 4a), DFMO treatment resulted in a significantly higher total polyamine content. Given that ornithine and arginine are interconvertible, the inhibition of ODC activity probably increased the availability of substrate for ADC, followed by the upregulation of ADC activity and thus higher total polyamine levels in the leaves. However, ADC cannot compensate for ODC in young developing tissues because there is less ADC activity in younger leaves (Paschalidis & Roubelakis‐Angelakis, 2005). Therefore, DFMO applied before flowering resulted in the strong inhibition of plant growth. Treatment with the plant growth regulator Ethephon^®^ reduced the polyamine content of the leaves but did not affect the amount of polyamines in the roots, thus explaining the lack of impact on the leaf phenotype. These data show that the finely tuned interaction of enzyme activities related to polyamine biosynthesis makes it difficult to control the total polyamine content and thus restore the NA leaf phenotype in the LA Burley 21 variety.

## 5 CONCLUSIONS

We have demonstrated the key role of polyamines in tobacco leaf maturation, offering valuable insight into the consequences of disrupting the equilibrium between the nicotine, polyamine, and ethylene pathways. Our data show that deleting the *Nic2* locus (HI variety) has no significant impact on the mesophyll cell number, chlorophyll levels and polyamine content, whereas deleting the *Nic1* locus (LI variety) significantly increases the cell number, chlorophyll levels and polyamine content in the leaves. In the LA variety (*nic1* ^*−*^ *nic2* ^*−*^), the loss of *Nic2* has no effect on the chlorophyll content, but amplifies the effect of deleting the *Nic1* locus on the upregulation of polyamine biosynthesis and increase in mesophyll cell number per unit leaf area. Treatment with polyamine biosynthesis inhibitors and Ethephon^®^ restored the polyamine content either in the roots (DFMO, DFMO/Ethephon^®^) or leaves (Ethephon^®^), but neither of the treatments achieved the complete amelioration of the LA leaf phenotype. If a treatment could reduce the polyamine content of LA Burley 21 plants in the roots and leaves simultaneously, the phenotype is likely to improve further. However, the LA Burley 21 phenotype is influenced by the loss of all genes in the *Nic1/Nic2* loci (more than seven genes). We found that the *Nic1* locus has a much greater impact on leaf maturation in LA Burley 21 than *Nic2*, therefore the characterization of the *Nic1* gene cluster will facilitate the development of novel strategies to manufacture high‐quality tobacco products from LA Burley 21 plants.

## AUTHORS CONTRIBUTION

DX, UW, ML, and JAS, conceived the original research plans; GN, DV, IC, MH, HS, and JF performed the experiments, GN, HS, and SS designed and supervised the experiments; DV, IC, and MH established and developed protocols, ML, JF, AA, CK, UW, DX, and JAS contributed reagents/materials/analysis tools, GN and IC analyzed and interpreted the data, GN, HS, SS, and ML wrote the article.
