# Supplementary material for: Polyamines delay leaf maturation in low‐alkaloid tobacco varieties
Source: Plant Direct. 2018 Jul 31;2(7):e00077. doi: 10.1002/pld3.77 (PMC6508808; doi:10.1002/pld3.77)
Supplement: Supplementary file 1 [file PLD3-2-e00077-s001.docx]

**Supplementary material**

Supplementary Table 1: Compound parameter for polyamine quantification. DP = declustering potential, CE = collision energy, CEP = collision cell entrance potential, CXP = collision cell exit potential, EP = entrance potential.

| **Dansylated Compound** | **Quantifier/ Qualifier** | **Parent mass [m/z]** | **Product mass [m/z]** | **DP [eV]** | **EP [eV]** | **CEP [eV]** | **CE [eV]** | **CXP [eV]** | **RT [min]** |
| --- | --- | --- | --- | --- | --- | --- | --- | --- | --- |
| Spermine | Quantifier | 1135.39 | 360.3 | 86 | 10 | 48 | 65 | 4 | 4.3 |
| Spermine | Qualifier | 1135.39 | 170.3 | 86 | 10 | 48 | 121 | 4 | 4.3 |
| Spermidine | Quantifier | 845.228 | 360.3 | 96 | 9.5 | 34 | 53 | 4 | 3.9 |
| Spermidine | Qualifier | 845.228 | 170.3 | 96 | 9.5 | 34 | 81 | 4 | 3.9 |
| Putrescine | Quantifier | 555.119 | 170.3 | 61 | 7.5 | 24 | 45 | 4 | 3.2 |
| Putrescine | Qualifier | 555.119 | 168.3 | 61 | 7.5 | 24 | 79 | 4 | 3.2 |
| Hexamethyldiamine | Quantifier | 583.14 | 170.3 | 70 | 10 | 28.809 | 50 | 4 | 3.5 |
| Hexamethyldiamine | Qualifier | 583.14 | 169.2 | 70 | 10 | 28.809 | 50 | 4 | 3.5 |
