# Supplementary material for: Polyamines delay leaf maturation in low‐alkaloid tobacco varieties
Source: Plant Direct. 2018 Jul 31;2(7):e00077. doi: 10.1002/pld3.77 (PMC6508808; doi:10.1002/pld3.77)
Supplement: Supplementary file 2 [file PLD3-2-e00077-s002.pdf]

Manuscript ID biot.201800170-T1

Dear Prof. Lawson,

Please find enclosed our revised manuscript with the title “Polyamines delay leaf maturation in low-alkaloid tobacco varieties” for submission to Plant Direct.
We thank the reviewers for their effort in reviewing our paper. We have revised our manuscript according to the revision instructions.
We hope that the revisions are satisfactory and fulfill the requirements for publication in the Plant Direct. If you have any further questions regarding our revised manuscript please do not hesitate to contact me.

I look forward to hearing from you,

Stefan Schillberg

Reviewer 1:

1*) The introduction is well written and extensively covers the background. However, some key facts about tobacco have been emitted from the WHO report. Given the subject area Line 55-58, deserve to be included.*

1, Tobacco kills up to half of its users.
2, Tobacco kills more than 7 million people each year. More than 6 million of those deaths are the result of direct tobacco use while around 890 000 are the result of non-smokers being exposed to second-hand smoke.
3, Around 80% of the world's 1.1 billion smokers live in low- and middle-income countries.

We agree with the reviewer and additional information on the subject is included in the introduction (lines 56-58). Regarding the more detailed information from the WHO report we refer to the corresponding reference (line 51 and 58).

Major Points
1, The first thing that struck me was the unusual use of the word 'Ripening' in the title referring to leaf development or leaf maturation. I've not seen this term used in this context as it generally refers to the structural a colour changes associated with fruit maturation. Recommend substituting ripening for 'maturation' in the case of leaf. Otherwise, it is essential that you better describe what is meant by 'leaf ripening' in this context.

The word *'*Ripening*'* has been replaced by the word *'*Maturation*'* in the revised manuscript.

Minor points
1, Lines 47. '7.4 million tons (FAOSTAT, 2014)' refers to the harvest data from a report from 2000. More up to date data can be found at
https://www.statista.com/statistics/261189/global-tobacco-production-since-1980/

In 2016, worldwide tobacco harvest was 6.6 million metric tonnes decreasing annually from 7.6 million metric tonnes in 2013.

Updated information on the global tobacco production plus the corresponding website has been included in the revised manuscript (line 47-48). The older FAOSTAT reference has been deleted from the cited literature.

2, Line 57 'development of tobacco cultivars with lower levels of nicotine, which could potentially yield tobacco products having a less public health impact' is quite misleading. Reducing nicotine content is unlikely to have any great influence on public health. Although pointing out that some tobacco-specific nitrosamines may be associated with tobacco use related cancers, this is likely minor compared with the other many tobacco carcinogenic agents and the toxins that come from burning the tobacco itself, including TAR and carbon monoxide which damage the cells that line the lungs.

We agree with the reviewer's comments and have modified the sentence accordingly (line 60-67). Please note that the sentence *'development of tobacco cultivars with lower levels of nicotine, which could potentially yield tobacco products having a less public health impact'* is based on the fact that the addictive effect of nicotine is mainly responsible for the widespread use of tobacco products. It has been postulated (Apelberg et al. (2018) Potential public health effects of reducing nicotine levels in cigarettes in the United States. N. Engl. J. Med. 378, 1725-1733) that development of tobacco cultivars with lower levels of nicotine would result in lower intake of nicotine, lower nicotine dependence and thus, to less health issues. The reference Apelberg et al (2018) plus the information and the corresponding website of the FDA advanced notice of proposed rulemaking (ANPR) describing the implementation of new standards for nicotine products have been included in the revised manuscript (lines 61-67).

Reviewer #2:

The manuscript by Nolke et al. describes the relationship between the impaired leaf ripening phenotype of tobacco plants possessing the nic1 and nic2 mutations that confer a low nicotine phenotype, and the accumulation of free and conjugated polyamines (PAs). A strong correlation was observed between increased PA content and delayed ripening which is consistent with the role that PAs have been shown to play in regulating senescence in model systems. Treatment of LA Burley 21 plants with inhibitors of PA synthesis and/or the senescence-inducing growth regulator Ethephon further supported the contention that increased PA accumulation in the LA materials is at least partially responsible for their poor ripening phenotype. The paper is very well written and provides very relevant insights into the mechanisms underlying this phenomenon that is of particular value given the recent increased interest in low nicotine tobaccos by the FDA and tobacco companies alike. I just have a couple of minor comments:

1. Line 62 - the way this sentence is written suggests that all nicotine produced in leaves is exuded by trichomes. Although this occurs, the accumulation and storage of nicotine in the vacuoles of leaf cells (many cell types, not just trichomes) is the predominant fate of the nicotine and should be indicated.

We agree with the reviewer's comment and changed the sentence in line 62 (line 71 in the revised manuscript) accordingly. A reference on nicotine accumulation in the vacuole of leaf cells has been added.

2. Line 73 - "can be used" should be changed to "has been used"

The wording ‘can be used’ in line 73 (line 82 in the revised manuscript) has been changed to ‘has been used’.
